# Nutrient restriction during late gestation reduces milk yield and mammary blood flow in lactating primiparous beef females

**DOI:** 10.1093/jas/skae016

**Published:** 2024-01-19

**Authors:** Colby A Redifer, Lindsey G Wichman, Abigail R Rathert-Williams, Erin M Shangraw, Thomas B McFadden, Allison M Meyer

**Affiliations:** Division of Animal Sciences, University of Missouri, Columbia, MO 65211, USA; Division of Animal Sciences, University of Missouri, Columbia, MO 65211, USA; Division of Animal Sciences, University of Missouri, Columbia, MO 65211, USA; Division of Animal Sciences, University of Missouri, Columbia, MO 65211, USA; Division of Animal Sciences, University of Missouri, Columbia, MO 65211, USA; Division of Animal Sciences, University of Missouri, Columbia, MO 65211, USA

**Keywords:** beef heifers, developmental programming, Doppler ultrasonography, lactation, mammary gland, nutrient partitioning

## Abstract

Fall-calving primiparous beef females [body weight (**BW**): 451 ± 28 (SD) kg; body condition score (**BCS**): 5.4 ± 0.7] were individually-fed 100% (control; CON; *n* = 13) or 70% (nutrient restricted; NR; *n* = 13) of estimated metabolizable energy and metabolizable protein requirements from day 160 of gestation to calving. Post-calving, all dams were individually-fed tall fescue hay supplemented to meet estimated nutrient requirements for maintenance, growth, and lactation until day 149 of lactation. Four-hour milk yields were collected on days 21, 42, 63, 84, 105, and 147 of lactation, and milk nutrient composition was determined. Doppler ultrasonography of both pudendoepigastric arterial trunks was conducted every 21 d from days 24 to 108 of lactation. Total mammary blood flow was calculated, and hemodynamics from both sides were averaged. Data were analyzed as repeated measures with nutritional plane, day of lactation, their interaction, calving date, and calf sex (if *P* < 0.25) as fixed effects. We previously reported that post-calving, NR dams weighed 64 kg less and were 2.0 BCS lower than CON, but calf birth weight was not affected. Milk weight and volume were 15% less (*P* = 0.04) for NR dams than CON. Milk protein concentration was lower (*P* = 0.008) for NR dams than CON, but triglyceride and lactose concentrations were not affected (*P* ≥ 0.20) by nutritional plane. Milk urea N concentration of NR dams tended to be greater (*P* = 0.07) on day 42 but was lower (*P* = 0.01) on day 147 of lactation than CON. Total milk protein, triglyceride, and lactose yields were less (*P* ≤ 0.05) for NR dams than CON. Total milk urea N yield was less (*P* ≤ 0.03) for NR dams than CON on days 21, 63, and 147 of lactation. Maternal heart rate was greater (*P* = 0.008), but pudendoepigastric arterial trunk peak systolic velocity, resistance index, and cross-sectional area were less (*P* ≤ 0.04) and pulsatility index tended to be less (*P* = 0.06) for NR dams than CON. Mammary blood flow was 19% less (*P* = 0.004) for NR dams than CON, but mammary blood flow relative to milk weight or dam BW was not affected (*P *≥ 0.14) by nutritional plane. Most milk yield, milk nutrient composition, and mammary blood flow variables were affected (*P *≤ 0.04) by day of lactation. In summary, first-parity beef females that were nutrient restricted during late gestation and then fed to meet estimated nutrient requirements during lactation had decreased milk nutrient yield and a similar reduction in mammary blood flow.

## Teaser

First-parity beef females that were nutrient restricted during late gestation and then fed to meet estimated nutrient requirements during lactation had 15% lower milk yield, reduced milk protein concentration, and less total milk nutrients available for their calves than control females. Similar to the decrease in milk production, previously nutrient restricted dams had 19% less mammary blood flow than controls.

## Introduction

Maternal energy and protein requirements increase dramatically across pregnancy in the beef female to support exponential growth of not only the uteroplacenta and fetus, but also the mammary gland (Ferrell et al., 1976; NASEM, 2016). Most mammary secretory cell formation to prepare for the upcoming lactation occurs during pregnancy (Davis, 2017). The first-parity beef female is still growing, and final prepartum mammary growth and differentiation are occurring for the first time. The concept that gestational nutrient restriction can decrease prenatal nutrient delivery to the fetus and developmentally-program postnatal growth, metabolic function, and productivity has been studied with some regularity (Wu et al., 2006; Reynolds et al., 2010). However, the idea that maternal nutrition during pregnancy can impair mammary development and future milk production, indirectly programming postnatal development by decreasing nutrient availability to the offspring pre-weaning, has been given far less attention. In primiparous ewes that were nutrient restricted during mid- and late gestation, prenatal and postnatal nutrient delivery were decreased independently of 1 another, suggesting that both contribute to decreased offspring growth in early life (Meyer et al., 2010, 2011). Milk production data from late gestational undernutrition in primiparous beef females is sparse, inconsistent, and measured at limited timepoints (Corah et al., 1975; Kroker and Cummins, 1979; Wiley et al., 1991), and physiological mechanisms, such as mammary blood flow during lactation, have rarely been studied.

Data reported here are from an intensive experiment to determine the effects of late gestational nutrient restriction in individually-fed first-parity beef females on prenatal and postnatal nutrient availability and utilization by their calves. We reported that maternal growth was slowed for nutrient restricted heifers during late gestation, and they mobilized maternal stores so that fetal growth was spared (Redifer et al., 2023). Additionally, nutrient restricted dams produced less colostrum, but it was more concentrated so that total colostral nutrients and immunoglobulins, except lactose, were similar (Redifer et al., 2023). We hypothesized that dams nutrient restricted during late gestation then fed to meet estimated nutrient requirements during lactation would have decreased milk production; thus, postnatal nutrient availability for their calves would be reduced. Mechanistically, we hypothesized that the reduction in milk yield would be partially explained by a decrease in blood flow supplying the mammary gland. Our objectives were to determine the effects of late gestational nutrient restriction on milk yield, milk nutrient composition, and mammary blood flow in primiparous beef females fed to meet estimated nutrient requirements during lactation.

## Materials and Methods

All procedures were approved by the University of Missouri Animal Care and Use Committee (Protocol #9877), and research was conducted at the University of Missouri Beef Research and Teaching Farm (Columbia, MO).

### Animal management and diets during gestation

Animal management and nutritional plane treatments applied during late gestation were described in Redifer et al. (2023). Briefly, 26 single-sired fall-calving Hereford × Simmental-Angus crossbred beef heifers [initial body weight (**BW**) = 451 ± 28 (SD throughout methods) kg, initial body condition score (**BCS**) = 5.4 ± 0.7, calving at 2 yr of age] bred to a single Angus sire were allocated by BW, BCS, fetal sex, and expected calving date to 1 of 2 late gestational nutritional planes from day 160 of gestation to parturition. Control (**CON**; *n* = 13) heifers were individually-fed 100% of estimated metabolizable energy (**ME**) and metabolizable protein (**MP**) requirements for maintenance, pregnancy, and growth, whereas nutrient restricted (**NR**; *n* = 13) heifers were individually-fed 70% of ME and MP requirements. Heifers were housed in 12 partially-covered 3.7 × 15.8 m pens (*n* = 2 or 3 per pen), penned by nutritional plane, and individually-fed via a Calan gate feeding system (Calan Broadbent Feeding System, American Calan, Northwood, NH).

Nutrient requirements were estimated using an expected calf birth weight of 34 kg and projected maternal average daily gain of 0.36 kg/d. Metabolizable energy for maintenance was based on data for heifers in confinement (0.138 Mcal ME/kg non-gravid BW^0.75^; Freetly and Hales, personal communication). The equation used for ME for conceptus was published by Freetly et al. (2005). Equations from NASEM (2016) were utilized for ME for gain and MP for maintenance, conceptus, and gain. Nutrient requirements were adjusted weekly using the most recent dam BW (recorded every 21 d) and day of gestation.

From days 160 to 265 of gestation, diets were based on ad libitum chopped sorghum sudan hay [1.74 Mcal ME/kg, 6.69% crude protein (**CP**), 72.0% neutral detergent fiber (**NDF**), 52.8% acid detergent fiber (**ADF**); dry matter (**DM**) basis]. Starting on day 266 of gestation, diets were transitioned to ad libitum chopped endophyte-infected tall fescue-based hay (1.90 Mcal ME/kg, 7.22% CP, 65.1% NDF, 43.2% ADF; DM basis) over a 3-d period to allow for less supplement to meet estimated nutrient requirements for the end of gestation and upcoming lactation. Using expected individual hay intakes (estimated from the past week’s hay intakes), heifers were supplemented daily with whole corn, dried distillers’ grains with solubles (**DDGS**), and soyhull pellets to meet their assigned nutritional plane. Supplement for each heifer was formulated and weighed individually. Dams had ad libitum access to water and a trace mineralized salt block (Big 6 Mineral Salt, Compass Minerals America Inc., Overland Park, KS). Beginning on day 274 of gestation, heifers were closely monitored 24 h per day by trained personnel to ensure the time of calving was observed. Peripartum dams were managed as described in Redifer et al. (2023).

### Animal management and diets during lactation

Animal management and diets during lactation were described in Redifer et al. (2024). Following parturition, late gestational nutritional planes were terminated. If calving occurred prior to 0900 h, then the day of calving was considered day 1 of lactation; if calving occurred after 0900 h, then the following day was day 1 of lactation. From days 1 to 149 of lactation, all dams remained in pens with the Calan gate feeding system and were individually-fed 100% of estimated ME and MP requirements for maintenance, lactation, and growth. During this time, calves had access to milk only, as they could not access their dams’ diets in the Calan gates.

During lactation, ME for maintenance and gain and MP for maintenance and gain were calculated using the same equations and projected maternal average daily gain as used for late gestation. The ME for lactation equation from Freetly et al. (2005) was used, which is based on a predicted milk yield equation with an expected peak milk yield of 7.05 kg/d at 10 wk of lactation for primiparous females. Equations from NASEM (2016) were utilized for MP for lactation, using the predicted milk yield equation from Freetly et al. (2005). Nutrient requirements were adjusted weekly using the most recent dam BW (recorded every 21 d) and day of lactation.

Lactational diets were based on ad libitum chopped endophyte-infected tall fescue-based hay (1.93 Mcal ME/kg, 8.00% CP, 63.6% NDF, 42.0% ADF; DM basis). Using expected individual hay intakes (estimated from the past week’s hay intakes), dams were supplemented daily with whole corn, DDGS, and soyhull pellets to meet their estimated requirements. Supplement for each dam was formulated and weighed individually. The transition to lactational diets (100% of estimated nutrient requirements) and increased supplement for NR dams took place over the first 4 d of lactation. Dams had ad libitum access to water and a trace mineralized salt block (Big 6 Mineral Salt, Compass Minerals America Inc.).

### Milk yield and nutrient composition

Four-hour milk yields were collected on days 21, 42, 63, 84, 105, and 147 of lactation, determined within ± 1 d (days 21, 42, and 63) or ± 2 d (days 84, 105, and 147) of the actual day of lactation. Before a dam was moved to the chute for the initial milking, all calves from the respective pen were placed in a pen without fenceline contact to prevent suckling for the 4-h milk accumulation period. Prior to initial milking, each female received 1.0 mL (20 USP) of oxytocin injected into the jugular vein to facilitate milk letdown. Immediately following oxytocin injection, the udder and teats were cleaned using infant care wipes. Dams were milked using a portable milking machine until milk stopped flowing from ≥ 1 teat(s). The milking cluster was removed, and each quarter was hand-stripped of residual milk until empty, with the time of completion recorded. Milk obtained during the initial milking was discarded. All initial milkings took place between 1500 and 1630 h and corresponded with determining dam BW (Redifer et al., 2024). Dams were returned to their pens with free access to water and the hay remaining from their morning feeding, but they remained separate from all calves. Each female was then milked 4 ± 0.08 h after the time of the conclusion of her initial milking, using the same protocol as described earlier. The milk collected from both machine-milking and hand-stripping was combined to determine 4-h milk yield. Milk volume was determined using 1,000-mL or 2,000-mL graduated cylinders, and milk was then weighed. Each dam’s 4-h milk weight and volume were multiplied by 6 to estimate daily milk production. Milking machine buckets, collection vessels, and graduated cylinders were rinsed thoroughly with water between each female and washed between milk yield days. The milk collected from each female was mixed well, and subsamples were aliquoted into multiple 2-mL microcentrifuge tubes and stored at −20°C until analysis.

Milk samples were analyzed for protein, triglycerides, lactose, and urea N as described by Rathert-Williams et al. (2023). Triglyceride results were used as a measure of milk fat, as they represent approximately 98% of lipids in milk (Walstra and Jenness, 1984). All samples were analyzed in duplicate with pooled controls and read on a UV-visible microplate reader (Biotek Synergy HT, Biotek Instruments Inc., Winooski, VT). The intraassay and interassay CV were 2.9% and 4.2% for protein, 3.6% and 3.4% for triglycerides, 2.4% and 2.7% for lactose, and 3.9% and 1.9% for urea N, respectively. Total milk nutrient yields were determined as nutrient concentration multiplied by calculated 24-h milk volume.

### Mammary blood flow

**Figure 1.**
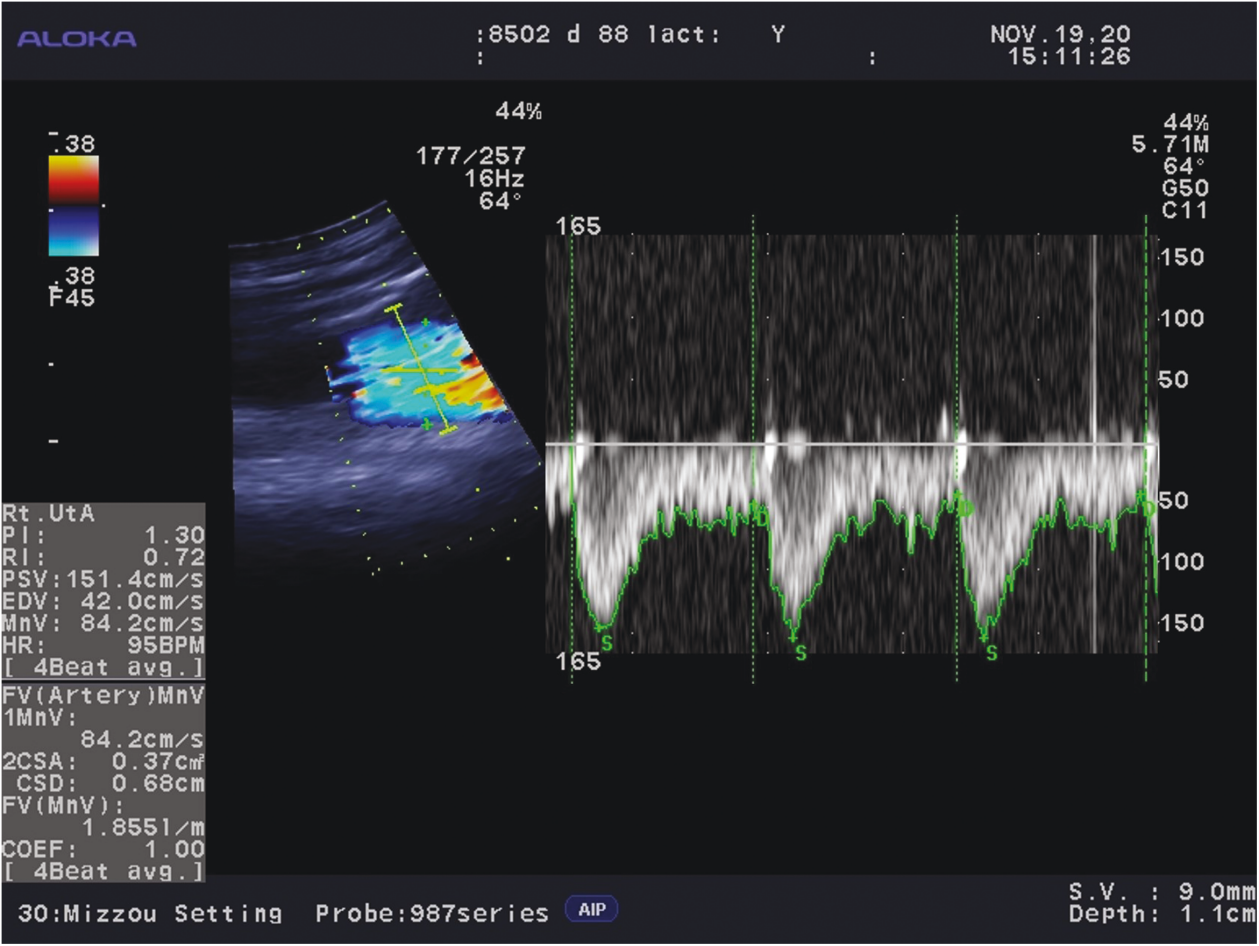
Color Doppler sonogram of the right pudendoepigastric arterial trunk of a primiparous beef female on day 88 of lactation. The image was obtained in B|D mode on an Aloka Prosound α6 machine after taking measurements chute-side. In B mode (left side), Doppler flow gain was set at 45, the angle of insonation (64°) was set parallel to the walls of the artery in a longitudinal view, and the caliper function was used to measure the cross-sectional diameter of the artery perpendicular to the angle of insonation. In D mode (right side), 4 representative and consistent cardiac waveforms were selected (only 3 are shown), from a similar set of frames as those from which the vessel was chosen. The machine calculated all variables, as displayed at the bottom left.

Mammary blood flow was measured 3 d after milk yield determination on days 24, 45, 66, 87, and 108 of lactation and was collected within ± 2 d (day 24), ± 1 d (days 45, 66, and 87), or ± 3 d (day 108) of the actual day of lactation. Mammary blood flow was assessed via transrectal color Doppler ultrasonography using an Aloka Prosound α6 (Hitachi Aloka Medical, Ltd, Wallingford, CT) equipped with a 7.5 MHz convex finger transducer (Aloka USD-995). Blood flow to the left and right pudendoepigastric arterial trunks was measured using methods similar to those described by Götze et al. (2010). The pudendoepigastric arterial trunk on each side splits into the caudal epigastric and external pudendal arteries, the latter of which is the main arterial blood supply to the mammary gland. With the ultrasound in B-mode with color Doppler, the probe was inserted through the rectum in a dorsal view to locate the abdominal aorta. The aorta was traced caudally to identify the external iliac artery branching off the internal iliac artery. A cross-sectional view of the external iliac artery was followed ventrally until it transitioned into the femoral artery and deep femoral artery. As the pair of arteries continued to be followed, the femoral artery quickly disappeared from view into the musculature. The deep femoral artery was traced further ventrally, and a few centimeters after the bifurcation with the femoral artery, the deep femoral artery branched into the medial circumflex femoral artery and the pudendoepigastric arterial trunk. Immediately posterior to this bifurcation, the finger transducer was oriented so that a longitudinal section of the pudendoepigastric arterial trunk could be visualized and maintained at an appropriate angle of insonation. The width of the sample gate was set slightly larger than the diameter of the pudendoepigastric arterial trunk. At that time, the ultrasound machine was then switched to a split screen view so that the longitudinal section of the artery (B-mode with color Doppler) and cardiac waves could be viewed simultaneously (Figure 1). After a consistent set of cardiac waves and a clear view of the vessel were obtained, the screen was paused so that data could be collected.

On each ultrasound day, both the left and right pudendoepigastric arterial trunks were measured. For both vessels, 3 or 4 separate ultrasound scans were obtained, and each scan included ≥ 2 cardiac cycle waveforms. If after 3 scans were conducted, data from 1 was inconsistent with the other 2, then a 4^th^ scan was conducted, and either all 4 scans were kept, or the inconsistent scan was removed. For all data collection, a single trained technician conducted the scans, and a separate single trained technician captured the measurements on the ultrasound machine, with all measurements made and data recorded chute-side. All ultrasound examinations were conducted between 1000 and 1600 h and lasted approximately 40 min for each female. The average angle of insonation for obtaining blood flow waveforms for each side of a single female was maintained between 57 and 70° (average: 63.1°). The average angle of insonation from past scans was used to maintain consistency of angle across all 5 timepoints for a female. Flow gain was set between 40 and 50 (average: 47.5). Maternal heart rate, peak systolic velocity, end diastolic velocity, mean velocity, pulsatility index, resistance index, cross-sectional diameter, cross-sectional area, and blood flow were recorded for each side separately. The machine software calculated pulsatility index = (peak systolic velocity − end diastolic velocity)/ mean velocity; resistance index = (peak systolic velocity − end diastolic velocity)/ peak systolic velocity; and blood flow (L/min) = mean velocity (cm/s) × cross-sectional area (cm^2^) × 60 (s/min)/ 1,000. Total mammary blood flow was calculated as the sum of the blood flow of the left and right pudendoepigastric arterial trunks. All other mammary blood flow variables were averaged for the 2 sides. Mammary blood flow relative to milk weight and dam BW were calculated using the milk weight and dam BW determined 3 d prior to a respective mammary blood flow measurement.

### Statistical analysis

One dam (CON) was completely removed from the study due to late gestational abortion. One calf (CON) was euthanized between days 105 and 147 of age due to severe weight loss and unthrifty appearance (diagnosed postmortem as renal dysplasia); thus, milk yield and composition data for its dam were not determined on day 147 of lactation. Mammary blood flow was not measured for 2 females (both CON) because abnormal vasculature made it difficult to get reliable data. Mammary blood flow was not determined on day 86 of lactation for 1 female (NR). This resulted in CON *n* = 11 or 12 and NR *n* = 13 for milk data and CON *n* = 10 and NR *n* = 12 or 13 for mammary blood flow data.

Milk yield, milk nutrients, and mammary blood flow were analyzed using the MIXED procedure in SAS 9.4 (SAS Institute Inc., Cary, NC) with late gestational nutritional plane, day of lactation, and their interaction as fixed effects and animal as the experimental unit. These were considered repeated measures using the majority best-fit covariance structure (based on Akaike Information Criterion, Bayesian Information Criterion, and corrected Bayesian Information Criterion) specific for each variable (chosen from unstructured, compound symmetry, heterogeneous compound symmetry, autoregressive, and heterogeneous autoregressive). For all measures, Julian date of calving and calf sex (if *P* ≤ 0.25) were included as covariates (fixed effects). The nutritional plane × day of lactation interaction affected (*P* = 0.003) average angle of insonation for mammary blood flow (greater angle for CON than NR at day 24 of lactation); thus, it was included as a covariate in the statistical models for flow-related variables (peak systolic velocity, end diastolic velocity, mean velocity, total mammary blood flow, and relative mammary blood flow). Significance was considered when *P* ≤ 0.05, and tendencies were considered when 0.05 < *P* ≤ 0.10. In the absence of interactions, main effects were reported. Means were separated using least significant difference and the same *P*-value thresholds.

Pearson correlation coefficients were determined between milk weight and total mammary blood flow (taken 3 d after a respective milk yield) with all animals combined and also separated by late gestational nutritional plane. Pearson correlation coefficients were also determined between milk weight at each timepoint and calf birth weight with all animals combined and separated by late gestational nutritional plane. The CORR procedure in SAS 9.4 was used, and the strength of the correlation coefficients was classified as weak (*r* = 0.20 to 0.39), moderate (*r* = 0.40 to 0.59), strong (*r* = 0.60 to 0.79), or very strong (*r* ≥ 0.80) when *P*-values were less than the thresholds described earlier.

## Results

### Milk yield, nutrient composition, and total nutrients

**Figure 2.**
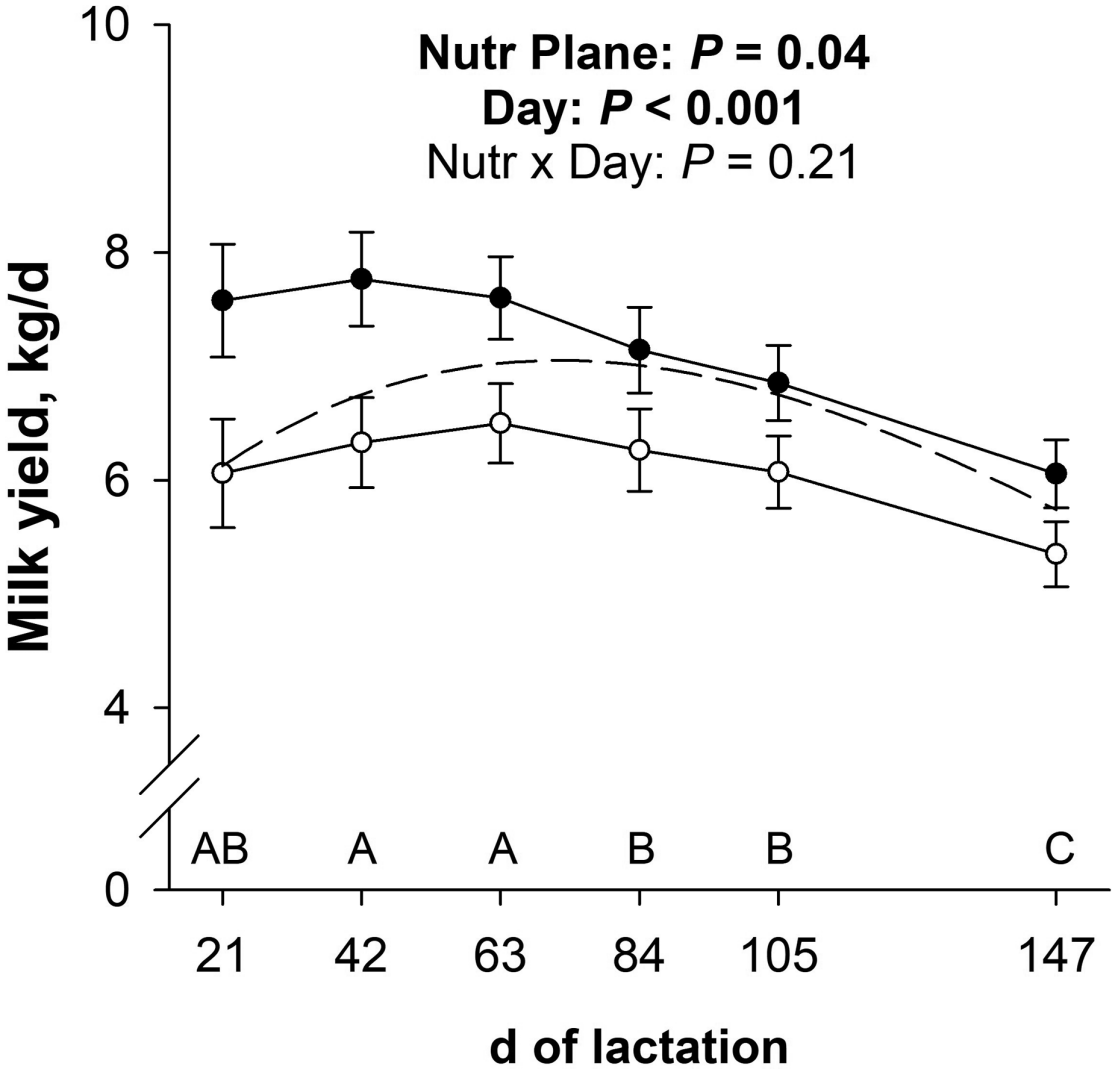
Effects of late gestational nutritional plane on milk weight from days 21 to 147 of lactation. Solid circles (●) represent primiparous beef females individually-fed 100% (Control; *n* = 11 or 12) and open circles (○) represent primiparous beef females individually-fed 70% (Nutrient Restricted; *n* = 13) of estimated metabolizable energy and metabolizable protein requirements for maintenance, pregnancy, and growth from day 160 of gestation to parturition. All dams were individually-fed 100% of estimated metabolizable energy and metabolizable protein requirements for maintenance, lactation, and growth from parturition until day 149 of lactation. Least squares means ± SEM are presented. ^A,B,C^Means for main effect of day differ (*P* ≤ 0.05). The predicted milk yield used to calculate estimated nutrient requirements for lactation is represented by the dashed line.

Milk weight and volume were not affected (*P* ≥ 0.21; Table 1; Figure 2) by the late gestational nutritional plane × day of lactation interaction. Milk weight and volume were 14.8 and 15.4% less (main effect; *P* = 0.04), respectively, for previously NR dams than CON. There was also an effect (*P* < 0.001) of day of lactation on milk weight and volume. Milk weight decreased (*P* ≤ 0.03) from days 63 to 84 and from days 105 to 147 of lactation. Milk volume decreased (*P* = 0.03) from days 63 to 84 of lactation, tended to decrease (*P* = 0.09) from days 84 to 105, and decreased (*P* < 0.001) from days 105 to 147 of lactation.

**Table 1.** Effects of late gestational nutritional plane and day of lactation of primiparous beef females on milk yield, nutrient composition, and total nutrients

Item	Late gestational nutritional plane^1^		SEM^3^	Day of lactation						SEM^3^	*P*-value^2^		
	CON	NR		21	42	63	84	105	147		Nutr	Day	Nutr × Day
Milk weight^4^, kg/d	7.16	6.10	0.35	6.82^ab^	7.05^a^	7.05^a^	6.70^b^	6.46^b^	5.71^c^	0.34	0.04	< 0.001	0.21
Milk volume^4^, L/d	7.10	6.01	0.35	6.73^abc^	6.97^ab^	6.99^a^	6.65^bc^	6.39^c^	5.62^d^	0.34	0.04	< 0.001	0.22
Nutrient composition													
Protein, g/dL	4.49	3.84	0.16	4.17^b^	3.74^c^	4.52^a^	4.41^ab^	3.41^c^	4.73^a^	0.23	0.008	< 0.001	0.81
Triglycerides, g/dL	2.76	2.64	0.10	2.40^c^	2.47^c^	2.55^bc^	2.83^ab^	2.91^a^	3.04^a^	0.13	0.35	< 0.001	0.41
Lactose, g/dL	4.52	4.58	0.04	4.53^abc^	4.60^a^	4.59^a^	4.57^ab^	4.51^bc^	4.48^c^	0.04	0.20	0.03	0.62
Milk urea N, mg/dL	—	—	—	—	—	—	—	—	—	—	0.16	< 0.001	0.02
CON	—	—	—	4.32^c^	4.55^c^	5.67^b^	5.77^b^	6.14^b^	9.05^a^	0.53			
NR	—	—	—	4.07^z^	5.11^y^^#^	5.15^y^	5.97^x^	6.24^wx^	7.21^w^*	0.47			
Total nutrients^5^													
Protein, g/d	316	226	12	278^bc^	256^c^	316^a^	292^ab^	218^d^	267^bc^	13	< 0.001	< 0.001	0.15
Triglycerides, g/d	195	155	9	159	170	181	186	185	170	10	0.004	0.14	0.37
Lactose, g/d	320	276	16	305^abc^	321^a^	320^a^	303^b^	288^c^	252^d^	16	0.05	< 0.001	0.35
Milk urea N, mg/d	—	—	—	—	—	—	—	—	—	—	0.009	< 0.001	0.06
CON	—	—	—	330^d^	353^cd^	430^b^	412^bc^	417^b^	531^a^	30			
NR	—	—	—	244^z^*	314^y^	326^xy^*	364^xy^	374^x^	377^x^*	27			

^1^Primiparous dams were individually-fed either 100% (Control; CON) or 70% (Nutrient Restricted; NR) of estimated metabolizable energy and metabolizable protein requirements for maintenance, pregnancy, and growth from day 160 of gestation to parturition. All dams were individually-fed 100% of estimated metabolizable energy and metabolizable protein requirements for maintenance, lactation, and growth from parturition until day 149 of lactation.

^2^Probabilities of difference for late gestational nutritional plane (Nutr), day of lactation (Day), and their interaction.

^3^Standard error of the mean for CON (*n* = 11 or 12) and NR (*n* = 13).

^4^Daily milk production was estimated using 4-h milk weight or volume multiplied by 6.

^5^Total milk nutrient yields were calculated as nutrient concentration multiplied by calculated 24-h milk volume.

^a,b,c,d^Means differ (*P* ≤ 0.05) for main effect of day (or for CON across days).

^w,x,y,z^Means differ (*P* ≤ 0.05) for NR across days.

^*^Within day, NR was less (*P *≤ 0.05) than CON.

^#^Within day, NR tended to be greater (0.05 < *P* ≤ 0.10) than CON.

The late gestational nutritional plane × day of lactation interaction did not affect milk protein, triglyceride, or lactose concentrations (*P* ≥ 0.41; Table 1). Previously NR dams had lower (main effect; *P* = 0.008) milk protein concentration than CON, but triglyceride and lactose concentrations were not affected (*P* ≥ 0.20) by nutritional plane. Day of lactation affected (*P* ≤ 0.03) milk protein, triglyceride, and lactose concentrations. Milk protein concentration decreased (*P* ≤ 0.005) from days 21 to 42 and from days 84 to 105 of lactation but increased (*P* < 0.001) from days 42 to 63 and from days 105 to 147 of lactation. Milk triglyceride concentration tended to increase (*P* = 0.08) from days 63 to 84 of lactation. Milk lactose concentration tended to increase (*P* = 0.08) from days 21 to 42 of lactation. Milk urea N concentration was affected (*P* = 0.02) by the late gestational nutritional plane × day of lactation interaction. On day 42 of lactation, milk urea N concentration tended to be greater (*P* = 0.07), but on day 147 of lactation urea N concentration was lower (*P* = 0.01), for NR dams than CON. For CON dams, milk urea N concentration increased (*P* < 0.001) from days 42 to 63 and from days 105 to 147 of lactation. For NR dams, milk urea N concentration increased (*P* ≤ 0.02) from days 21 to 42 and from days 63 to 84 of lactation and tended to increase (*P* = 0.06) from days 105 to 147 of lactation.

Total milk protein, triglyceride, and lactose yields were not affected (*P* ≥ 0.15; Table 1) by the late gestational nutritional plane × day of lactation interaction. Previously NR dams yielded 28.5% less (main effect; *P* < 0.001) total milk protein, 20.5% less (main effect; *P* = 0.004) total milk triglycerides, and 13.8% less (main effect; *P* = 0.05) total milk lactose than CON. Additionally, day of lactation affected (*P* < 0.001) total milk protein and lactose yields but did not affect (*P* = 0.14) total milk triglyceride yield. Total milk protein yield increased (*P* < 0.001) from days 42 to 63 of lactation, tended to decrease (*P* = 0.10) from days 63 to 84, decreased (*P* < 0.001) from days 84 to 105, and increased (*P* = 0.001) from days 105 to 147 of lactation. Total milk lactose yield decreased (*P* ≤ 0.05) from days 63 to 147 of lactation. There tended to be a late gestational nutritional plane × day of lactation interaction (*P* = 0.06) for total milk urea N yield. Total milk urea N yield was less (*P* ≤ 0.03) for NR dams than CON on days 21, 63, and 147 of lactation. For CON dams, total milk urea N yield increased (*P* ≤ 0.01) from days 42 to 63 and from days 105 to 147 of lactation. For NR dams, total milk urea N yield increased (*P* = 0.02) from days 21 to 42 of lactation.

### Mammary blood flow

The late gestational nutritional plane × day of lactation interaction did not affect (*P* ≥ 0.17; Table 2) any mammary blood flow variables; thus, only the main effects of nutritional plane and day of lactation were considered. Previously NR dams had a greater (main effect; *P* = 0.008) maternal heart rate than CON. Maternal heart rate was also affected (*P* < 0.001) by day of lactation, as it decreased (*P* = 0.05) from days 66 to 87 of lactation.

**Table 2.** Effects of late gestational nutritional plane and day of lactation of primiparous beef females on mammary blood flow during lactation

Item	Late gestational nutritional plane^1^		SEM^3^	Day of lactation					SEM^3^	*P*-value^2^		
	CON	NR		24	45	66	87	108		Nutr	Day	Nutr × Day
Maternal heart rate^4^, beats/min	81.3	86.2	1.2	85.2^a^	87.1^a^	84.7^a^	81.7^b^	80.0^b^	1.3	0.008	< 0.001	0.92
Peak systolic velocity^4^, cm/s	112	102	3	110^ab^	113^a^	106^b^	103^b^	102^b^	3	0.03	0.04	0.17
End diastolic velocity^4^, cm/s	28.1	29.0	1.0	30.7^a^	30.0^ab^	27.9^bc^	27.3^c^	26.7^c^	1.0	0.51	0.007	0.88
Mean velocity^4^, cm/s	59.1	55.9	1.6	61.0^a^	60.7^a^	56.4^b^	55.1^b^	54.3^b^	1.8	0.15	0.006	0.54
Pulsatility index^4^	1.40	1.31	0.04	1.28^b^	1.35^a^	1.37^a^	1.38^a^	1.39^a^	0.03	0.06	0.008	0.52
Resistance index^4^	0.746	0.711	0.009	0.716	0.730	0.734	0.734	0.737	0.008	0.02	0.11	0.68
Cross-sectional area^4^, cm^2^	0.574	0.489	0.030	0.507^c^	0.564^a^	0.524^bc^	0.544^ab^	0.517^c^	0.022	0.04	< 0.001	0.30
Total mammary blood flow^5^, L/min	4.07	3.28	0.18	3.71^b^	4.11^a^	3.54^bc^	3.61^bc^	3.40^c^	0.15	0.004	< 0.001	0.64
Blood flow relative to milk weight^6^, L·min^−1^·kg^−1^·d^−1^	0.573	0.534	0.047	0.585^abc^	0.600^a^	0.506^c^	0.549^b^	0.527^bc^	0.050	0.54	0.02	0.36
Blood flow relative to dam BW^6^, mL·min^−1^·kg^−1^	7.84	7.09	0.38	7.88^b^	8.49^a^	7.13^c^	7.17^c^	6.65^c^	0.32	0.14	< 0.001	0.46

^1^Primiparous dams were individually-fed either 100% (Control; CON) or 70% (Nutrient Restricted; NR) of estimated metabolizable energy and metabolizable protein requirements for maintenance, pregnancy, and growth from day 160 of gestation to parturition. All dams were individually-fed 100% of estimated metabolizable energy and metabolizable protein requirements for maintenance, lactation, and growth from parturition until day 149 of lactation.

^2^Probabilities of difference for late gestational nutritional plane (Nutr), day of lactation (Day), and their interaction.

^3^Standard error of the mean for CON (*n* = 10) and NR (*n* = 12 or 13).

^4^Represents the average value from the left and right pudendoepigastric arterial trunks.

^5^Sum of mammary blood flow from the left and right pudendoepigastric arterial trunks.

^6^Milk weight and dam body weight (BW) determined 3 d prior to each respective total mammary blood flow measurement.

^a,b,c^Means differ (*P* ≤ 0.05) for main effect of day.

Peak systolic velocity was lower (main effect; *P* = 0.03; Table 2) for previously NR dams than CON; however, end diastolic velocity and mean velocity were not affected (*P* ≥ 0.15) by late gestational nutritional plane. Peak systolic velocity, end diastolic velocity, and mean velocity were affected (*P* ≤ 0.04) by day of lactation. Peak systolic velocity and mean velocity both decreased (*P* ≤ 0.03) and end diastolic velocity tended to decrease (*P* = 0.07) from days 45 to 66 of lactation. Previously NR dams tended to have a lower (main effect; *P* = 0.06) pulsatility index and had a lower (main effect; *P* = 0.02) resistance index than CON. Day of lactation also affected (*P* = 0.008) pulsatility index, as it increased (*P* = 0.03) from days 24 to 45 of lactation. Resistance index was not affected (*P* = 0.11) by day of lactation. Vessel cross-sectional area was less (main effect; *P* = 0.04) for NR dams than CON dams. Day of lactation also affected (*P* < 0.001) cross-sectional area. Cross-sectional area increased (*P* < 0.001) from days 24 to 45 of lactation, decreased (*P* < 0.001) from days 45 to 66, tended to increase (*P* = 0.06) from days 66 to 87, and decreased (*P* = 0.01) from days 87 to 108 of lactation.

**Figure 3.**
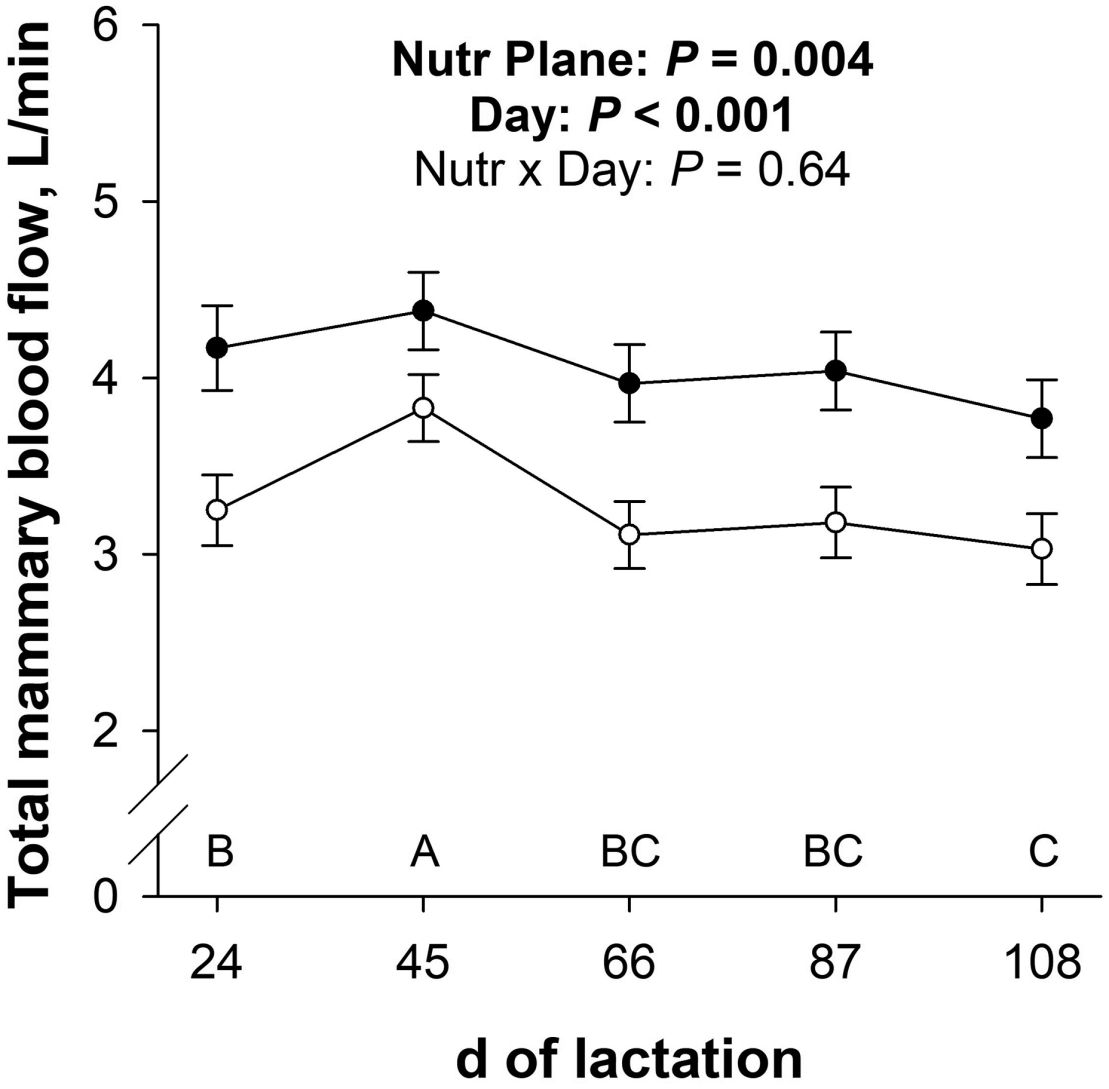
Effects of late gestational nutritional plane on total mammary blood flow from days 24 to 108 of lactation. Solid circles (●) represent primiparous beef females individually-fed 100% (Control; *n* = 10) and open circles (○) represent primiparous beef females individually-fed 70% (NR; *n* = 12 or 13) of estimated metabolizable energy and metabolizable protein requirements for maintenance, pregnancy, and growth from day 160 of gestation to parturition. All dams were individually-fed 100% of estimated metabolizable energy and metabolizable protein requirements for maintenance, lactation, and growth from parturition until day 149 of lactation. Least squares means ± SEM are presented. ^A,B,C^Means for main effect of day differ (*P* ≤ 0.05).

Previously NR dams had 19.4% less (main effect; *P* = 0.004; Table 2; Figure 3) total mammary blood flow than CON; however, mammary blood flow relative to milk weight or dam BW was not affected (*P* ≥ 0.14) by late gestational nutritional plane. Total mammary blood flow, mammary blood flow relative to milk weight, and mammary blood flow relative to dam BW were all affected (*P* ≤ 0.02) by day of lactation. Total mammary blood flow and mammary blood flow relative to dam BW increased (*P* ≤ 0.03) from days 24 to 45 of lactation and decreased (*P* < 0.001) from days 45 to 66 of lactation. Mammary blood flow relative to dam BW also tended to decrease (*P* = 0.06) from days 87 to 108 of lactation. Mammary blood flow relative to milk weight decreased (*P* = 0.001) from days 45 to 66 of lactation and increased (*P* = 0.03) from days 66 to 87 of lactation.

### Relationship of milk yield with mammary blood flow and calf birth weight

With CON and NR dams combined, milk weight had no relationship (*r* = −0.08 to 0.31; *P *≥ 0.16) with total mammary blood flow (taken 3 d after a respective milk yield) at any of the 5 timepoints. There was also no relationship between milk weight and total mammary blood flow when CON dams (*r* = −0.47 to 0.003; *P *≥ 0.17) and NR dams (*r* = −0.40 to 0.24; *P *≥ 0.17) were evaluated separately.

We reported that calf birth weight was not affected (*P* = 0.72; 29.9 vs. 30.7 ± 1.7 kg) by late gestational nutritional plane (Redifer et al., 2023). With CON and NR dams combined, calf birth weight had a moderate relationship (*r* = 0.40 to 0.53; *P *≤ 0.05) with milk weight on days 21, 42, 63, 84, and 105 of lactation, but calf birth weight was not correlated (*r = *0.30; *P* = 0.14) with milk weight on day 147 of lactation. For CON dams, calf birth weight had no relationship (*P* ≥ 0.35; Table 3) with milk weight at any timepoint during the first 147 d of lactation. For NR dams, calf birth weight had a very strong positive correlation (*P* < 0.001; Table 3) with milk weight on day 42 of lactation and strong positive correlations (*P* ≤ 0.03) with milk weight on days 21, 63, and 84 of lactation. Additionally, calf birth weight and milk weight on day 105 of lactation had a moderate positive correlation (*P* = 0.05) in NR dams, but calf birth weight was not correlated (*P* = 0.15) with milk weight on day 147 of lactation.

**Table 3.** Partial correlation coefficients (*r*) and associated *P*-values between milk yield and calf birth weight for each late gestational nutritional plane^1^

Milk yield, kg/d	Calf birth weight^2^, kg	
	CON	NR
Day 21	0.18 (*P* = 0.58)	0.60 (*P* = 0.03)
Day 42	0.30 (*P* = 0.35)	0.82 (*P* < 0.001)
Day 63	0.15 (*P* = 0.64)	0.75 (*P* = 0.003)
Day 84	0.16 (*P* = 0.63)	0.72 (*P* = 0.006)
Day 105	0.24 (*P* = 0.45)	0.55 (*P* = 0.05)
Day 147	0.21 (*P* = 0.54)	0.43 (*P* = 0.15)

^1^Primiparous dams were individually-fed either 100% (Control; CON; *n* = 11 to 12) or 70% (Nutrient Restricted; NR; *n* = 13) of estimated metabolizable energy and metabolizable protein requirements for maintenance, pregnancy, and growth from day 160 of gestation to parturition. All dams were individually-fed 100% of estimated metabolizable energy and metabolizable protein requirements for maintenance, lactation, and growth from parturition until day 149 of lactation.

^2^Calves were weighed at 0.9 ± 0.3 h (SD) of age (pre-suckling).

## Discussion

We reported that nutrient restricted dams weighed 63.6 kg less and were 2.0 BCS lower than controls post-calving, but calf BW and size at birth were not affected by late gestational nutrient restriction (Redifer et al., 2023). Nutrient restricted dams produced 40% less colostrum, yet it was generally more concentrated so that total colostral nutrients and immunoglobulins, except lactose, were similar (Redifer et al., 2023). While being individually-fed to meet estimated nutrient requirements during lactation, previously nutrient restricted dams recovered quickly metabolically, experienced compensatory growth, and replenished basal adipose reserves, but still had less body condition at weaning than controls (Redifer et al., 2024). In the current study, first-parity beef females that were previously nutrient restricted had less mammary blood flow than controls and prioritized partitioning nutrients to maternal growth and energy reserves instead of to lactation, both of which likely contributed to the reduction in milk yield and milk nutrients available to calves.

### Milk yield, nutrient composition, and total nutrients

Our findings strongly demonstrate that late gestational nutrient restriction can indirectly program postnatal calf growth and development by decreasing pre-weaning nutrient availability. Milk production was 15% less and milk protein concentration was lower even when all dams were individually-fed to meet estimated energy and protein requirements during the first 5 mo of lactation. Despite relatively minimal changes in milk nutrient concentrations, the substantial decrease in milk yield drove the reduction in total milk protein, triglycerides, lactose, and urea N available for consumption by calves born to nutrient restricted dams.

Late gestational undernutrition did not affect milk production in individually-fed beef heifers (Corah et al., 1975) but tended to reduce milk yield in individually-fed beef cows (Kennedy et al., 2019); however, both studies determined milk production at only 1 timepoint. In late gestational nutrient restriction studies in which beef heifers were managed in groups, milk yield was decreased (Ryley and Gartner, 1962; Kroker and Cummins, 1979) or not affected (Wiley et al., 1991). Mature beef cows grazing dormant Sandhills winter range during late gestation produced less milk than cows grazing corn crop residue (Larson et al., 2009). However, when 3-yr old beef cows were intentionally managed to calve at a BCS of 4 or 6, milk production was not affected by the decrease in maternal energy reserves available (Lake et al., 2005). In other studies, late gestational protein restriction (Valiente et al., 2018) or a high–low nutritional plane switchback design in late gestation (Anderson et al., 1981) did not affect milk production in beef females. Similar to our results, mid- and late gestational nutrient restriction in primiparous ewes resulted in 22% less milk production (Meyer et al., 2011).

In agreement with the current results, beef cows that calved at a BCS of 4 had lower milk protein concentration than cows that calved at a BCS of 6, while fat and lactose concentrations were not affected (Lake et al., 2005). Additionally, late gestational undernutrition in beef heifers reduced milk protein and fat concentrations but did not alter lactose concentration (Wiley et al., 1991). Other studies investigating late gestational nutrient restriction reported no effect on milk nutrient concentrations (Ryley and Gartner, 1962; Valiente et al., 2018; Kennedy et al., 2019). None of these studies provided milk nutrient yields, but presumably when milk yield differences existed, they drove divergence in total milk nutrients available to calves, much like we observed. Mid- and late gestational nutrient restriction in primiparous ewes resulted in decreased milk urea N concentration and less total milk lactose, protein, and urea N yields, but interestingly, milk protein concentration was greater (Meyer et al., 2011).

There are multiple factors that could account for the discrepancies in milk yield and nutrient composition outcomes, including the length and severity of undernutrition, lactation nutrient intake, genetic potential for milk production across populations, stage of lactation, frequency of sampling, parity, and methodologies used. Of the beef cattle studies cited earlier, only Kroker and Cummins (1979) and Valiente et al. (2018) measured milk production more than 3 times during lactation. The studies included both machine-milking (Ryley and Gartner, 1962; Wiley et al., 1991; Lake et al., 2005; Valiente et al., 2018; Kennedy et al., 2019) and weigh–suckle–weigh (Corah et al., 1975; Anderson et al., 1981; Larson et al., 2009) data, with milk accumulation durations ranging from 4 to 24 h. It has been demonstrated that both collection method (Mondragon et al., 1983; Beal et al., 1990; Albertini et al., 2012) and accumulation time (Ryley and Gartner, 1962) can affect milk yield estimation. Milk nutrient composition results can also be influenced by the analytical methods chosen. Most papers that reported milk composition used Fourier transform infrared spectroscopy analysis calibrated with dairy milk (Wiley et al., 1991; Lake et al., 2005; Kennedy et al., 2019), which we have shown may not give accurate results for beef milk samples compared with the colorimetric methods we utilized (Rathert-Williams et al., 2023).

Milk yields obtained from machine-milking represent milk production, but a calf may not be able to consume all of the milk produced. Yields derived from weigh–suckle–weigh methods are indicative of calf milk consumption, which may be limited by milk production in low-producing dams but may underestimate milk production in high-producing dams. Three-week old dairy calves of a similar weight as our calves at that age consumed more than 10 kg/d of milk replacer (Jasper and Weary, 2002; Schäff et al., 2016). This clearly suggests that calves born to dams of both late gestational nutritional planes in our study could likely consume all milk produced; therefore, these estimated yields are representative of daily milk production when calves are suckling naturally.

### Physiological factors affecting milk production

#### Lactational nutrient partitioning

The data presented here and in Redifer et al. (2024) illustrate that late gestational nutrient restriction in beef heifers altered nutrient partitioning during lactation. Previously nutrient restricted dams, when fed to meet estimated nutrient requirements during lactation, prioritized nutrient use for maternal growth and energy reserves over milk production during this compensatory period. Similar to the current study, primiparous ewes nutrient restricted during gestation experienced greater lactational average daily gain, even when consuming less dry matter intake (Meyer et al., 2010), and had lower milk production (Meyer et al., 2011). The lack of circulating glucose, triglyceride, and non-esterified fatty acid differences between dams from the late gestational nutritional planes after day 21 of lactation (Redifer et al., 2024) suggests that the lesser milk production was not a result of limited nutrient availability in circulation. Milk production of our previously nutrient restricted dams was lower than the expected milk yield curve of Freetly et al. (2005) after day 21 of lactation. This resulted in overestimation of lactational nutrient requirements; thus, additional nutrients were likely available for growth despite previously nutrient restricted dams still having less body condition than controls at weaning (Redifer et al., 2024).

Milk yields of control dams prior to day 84 of lactation were greater than the expected milk yield curve of Freetly et al. (2005), and lactational nutrient requirements were underestimated. If control dams were fed at a greater nutritional plane during lactation, then it is possible that they would have had greater milk production, and the shape of their lactation curve may have differed from that observed. Lactational circulating non-esterified fatty acid concentrations were low (approximately 100 µEq/L), yet control dams decreased in BCS (−0.35) and backfat thickness (−0.05 cm) during the first 5 mo of lactation (Redifer et al., 2024). While control dams achieved the targeted maternal growth during individual feeding, the mobilization of adipose reserves likely made up for the discrepancy between dietary nutrients provided for expected milk yield and nutrients required for the greater than expected milk production observed.

#### Mammary blood flow

Mammary substrate availability for milk synthesis largely depends on the rate of blood perfusing the mammary gland and the concentration of substrates in the blood (Davis and Collier, 1985). Doppler ultrasonography of the pudendoepigastric trunks provides a non-invasive, repeatable method to measure mammary blood flow (Piccione et al., 2004; Götze et al., 2010). In the current study, dams that were nutrient restricted during late gestation then fed to meet estimated nutrient requirements during lactation had 19% less mammary blood flow during the first 108 d of lactation, similar to the 16% lower milk yield observed through day 105 of lactation.

Lower mammary blood flow observed for previously nutrient restricted dams was due to smaller vessel cross-sectional area rather than lower mean velocity. It is possible that the slowed maternal growth in primiparous females during late gestational undernutrition affected systemic vessel size and whole-body blood volume. Conversely, late gestational undernutrition reduced mammary blood flow during pregnancy on 1 side in Kennedy et al. (2019), driven by lower mean velocity rather than smaller vessel size. The reduction in mammary blood flow of nutrient restricted dams was accompanied by greater pulsatility index in Kennedy et al. (2019); however, our nutrient restricted dams had lower pulsatility and resistance indices. Pulsatility and resistance indices represent measures of vascular resistance; thus, smaller, but seemingly more compliant, vessels were an unexpected result in the current study.

Few studies have characterized mammary blood flow in beef cattle, and those that have largely focused on mammary hemodynamics during gestational mammary growth rather than lactation. Nutrient restriction during early and mid-gestation (Silva et al., 2022), mid- and late gestation (Tanner et al., 2023), or late gestation (Mordhorst et al., 2017; Kennedy et al., 2019) has sometimes altered gestational mammary blood flow when evaluated by side (relative to the ipsilateral or contralateral uterine horn) but did not affect total mammary blood flow. The previously nutrient restricted cows in Kennedy et al. (2019) tended to have less milk yield at day 44 of lactation, but this was not accompanied by mammary blood flow differences. All of these studies were in mature beef cows that had successfully lactated before. Perhaps mammary gland development of first-parity beef females is more susceptible to gestational undernutrition because it is developing for the first time, which may explain the reduction in total mammary blood flow that we observed.

Previously nutrient restricted dams had an increased heart rate during lactation relative to controls. During late gestation, nutrient restricted dams had decreased heart rate compared with controls (unpublished data); thus, it appears that the greater heart rate during lactation may be a compensatory response to realimentation following restriction. In dairy cows, Thomas and Moore (1951) observed that when energy intake was changed, heart rate changed in the same direction, suggesting that heart rate is related to nutritional plane.

Many reviews on lactational physiology state that mammary blood flow and milk yield are positively related (Tucker, 1981; Davis and Collier, 1985; Svennersten-Sjaunja and Olsson, 2005), and utilizing Doppler ultrasonography to measure mammary blood has resulted in positive correlations with milk yield in dairy cows (Honnens et al., 2007; Götze et al., 2010; Berger et al., 2016). To our knowledge, this relationship has not been reported in beef cattle or primiparous females, but mammary blood flow was not correlated with milk weight at any timepoint in our study. While milk yield and mammary blood flow are closely related, it is unclear if milk production potential is the result of changes in mammary blood flow or elicits an adaptive response in mammary blood flow (Wall and McFadden, 2012). The values we observed across lactation for mammary blood flow relative to milk weight (0.51 to 0.60 L·min^−1^·kg^−1^·d^−1^) were slightly lower, but comparable to, those reported for early lactation dairy cows (0.66 to 0.81 L·min^−1^·kg^−1^·d^−1^; Götze et al., 2010).

Studies in dairy cattle reported that milking or oxytocin administration increased mammary blood flow (Davis and Collier, 1985; Gorewit et al., 1989). For dairy cows milked at regular intervals and with no calf at side, it is possible to ensure that mammary blood flow measurements are taken at a similar time relative to complete emptying of the udder for all cows, but this was not possible in the current study due to the intensive nature of data collection and the breadth of measures. While consideration was taken to conduct blood flow measurements for all animals during a similar time of day (1000 to 1600 h), calves being reared naturally by their dam did not allow for control of suckling time relative to blood flow measurements. It is possible that this partially explained the lack of correlation despite treatment means having a similar magnitude of difference.

#### Other physiological factors

Mammary growth in preparation for lactation is largely complete at parturition, with exponential mammary cell proliferation occurring during late gestation (Davis, 2017), yet little is known about how gestational nutrient restriction alters mammary development in beef cattle. Gestational undernutrition in ruminants has reduced mammary gland weight, increased percentage of alveolar luminal area (Swanson et al., 2008), and decreased alveoli per area (Neville et al., 2013), but no differences in percent proliferating alveolar cells, mammary gland cellularity, capillary area density, or angiogenic factor mRNA have been observed (Swanson et al., 2008; Vonnahme et al., 2011; Neville et al., 2013; Silva et al., 2022). In the current study, we did not determine if late gestational nutrient restriction affected mammary gland growth or development because dams needed to naturally raise their calves for further objectives, and these are best measured after slaughter. Additionally, it is unknown if mammary gland development, function, or blood flow changes occurring during 1 pregnancy or lactation are permanent or can recover in the following parity.

We observed moderate to very strong positive relationships between calf birth weight and milk weight from days 21 to 105 of lactation in previously nutrient restricted dams, but no relationship in control dams. Gestational mammary development and lactational mammary function are both under strict endocrine regulation by reproductive and metabolic hormones (Svennersten-Sjaunja and Olsson, 2005), which can be influenced by nutrition. The bovine placenta synthesizes large quantities of hormones such as estrone, progesterone, and placental lactogen (Anthony et al., 1995; Schuler et al., 2008). In an induced lactation model in dairy heifers, recombinant placental lactogen stimulated mammary growth and differentiation (Byatt et al., 1994) and increased milk production (Byatt et al., 1997). Yet, normal concentrations of placental lactogen in maternal circulation are quite low during pregnancy in cattle, and the physiological mechanism behind its action is poorly understood (Forsyth, 1986). The relationships we observed likely indicate that in the face of limited nutrient availability, there is synchrony in protecting fetal growth and similar levels of milk production, perhaps mediated through greater placental lactogen produced from larger placentas associated with greater fetal growth (Bauman and Currie, 1980; Forsyth, 1986).

### Lactation curve and day of lactation effects

In the current study, milk weight decreased from days 63 to 84 and again from days 105 to 147 of lactation. The predicted milk yield equation from Freetly et al. (2005) used to determine ME and MP requirements for lactation is based on an expected peak milk yield of 7.05 kg/d for primiparous females at 10 wk of lactation. Our control females had a numerical peak milk yield of 7.77 kg/d at day 42 of lactation, and the nutrient restricted females had a numerical peak milk yield of 6.50 kg/d at day 63 of lactation. While the milk yield curve of Freetly et al. (2005) underestimated control dam lactational nutrient requirements and overestimated nutrient restricted dam requirements, its curve shape was closer to that observed in the current study than the milk yield curve in NASEM (2016). Using a projected peak milk yield of 7.05 kg/d and the milk yield curve in NASEM (2016), lactational nutrient requirements would have been underestimated for control females at all timepoints and underestimated for nutrient restricted females at the early and late timepoints. In the current study, primiparous females from both late gestational nutritional planes had fairly persistent milk production from days 21 to 147 of lactation compared with NASEM (2016), but yield would have likely dwindled if sampled later in lactation. The observed milk yield persistence may be due to the relatively low milk production or primiparity of these females (Notter et al., 1978; Mondragon et al., 1983).

All mammary blood flow variables, except resistance index, were affected by day of lactation in the current study. In dairy cows, mammary blood flow appears to peak around the time of parturition, perhaps as blood is redirected from the uteroplacenta to the mammary gland, remains elevated during high milk production of early and mid-lactation, and steadily declines with decreasing milk production until dry off (Honnens et al., 2007; Götze et al., 2010). In the current study, vessel cross-sectional area and total mammary blood flow increased to their peak on day 45 of lactation, coinciding with numerical peak milk yield for controls. Surprisingly, most mammary blood flow variables decreased from days 45 to 66 of lactation, while the numerical peak milk yield for previously nutrient restricted dams did not occur until day 63 of lactation.

### Practical implications of decreased milk production

For the first 5 mo of life, calves had access to milk only, preventing compensation of decreased milk nutrient availability by consuming supplementary feed or forage. In commercial beef cow–calf production, pre-weaning calves may have limited opportunity to consume non-milk nutrients, if fall-born calves have minimal standing forage available over winter or cow–calf pairs are managed in a drylot setting. Additionally, utilization of poor-quality forage by pre-weaning calves can be low in traditional grazing settings. Calf size at birth was not affected by late gestational nutritional plane, but calf BW diverged by day 42 of age, and calves born to nutrient restricted dams weighed 15% less after 5 mo of consuming milk only (Redifer et al., 2024). After this point, cow–calf pairs were managed in drylots as groups where calves had access to ad libitum hay. Even with forage consumption, calves born to nutrient restricted dams still weighed 13% less at weaning (Redifer et al., 2024), largely attributed to the reduction in milk nutrients available. Lower milk production not only decreased weaned calf value, but it may also have negative implications on long-term production.

The reduction in nutrients partitioned to milk production, while negative for the calf, was likely of benefit to the dam and her chances of remaining in the herd the subsequent year. Previously nutrient restricted dams partitioned nutrients to compensatory maternal growth and replenishing basal adipose reserves instead of towards maximal milk production (Redifer et al., 2024), which likely allowed them to prioritize resumption of estrus and fertility (Randel, 1990; Hess et al., 2005) and allowed for acceptable reproductive performance (Redifer et al., 2024). In a production setting, improved postpartum nutrition may not occur during droughts or times of poor forage nutrient availability. Moreover, it is unlikely that lactational nutrition would improve so drastically immediately after calving; therefore, a greater insult to milk production or reproductive performance could be expected than that observed in the current study. In mature beef cows, 210-d total milk production linearly increased with increasing lactational energy intakes (Jenkins and Ferrell, 1992). Primiparous beef heifers that were nutritionally managed to calve at a BCS of 4.0 and then fed a low-energy diet had a 17% decrease in milk yield compared with those fed to maintenance during lactation (Lalman et al., 2000). Furthermore, postpartum interval to resumption of cyclicity linearly decreased with increasing energy intake (Lalman et al., 2000).

## Conclusions

In summary, these data illustrate the detrimental effects that late gestational nutrient restriction in first-parity beef females can have on milk production even when lactating dams are individually-fed to meet estimated nutrient requirements for maintenance, growth, and lactation during the first 5 mo of lactation. The altered nutrient partitioning due to gestational undernutrition and the reduction in mammary blood flow during lactation likely contributed to the decreased milk yield observed in previously nutrient restricted dams. While shifting nutrients away from milk production and towards replenishing body stores likely benefitted rebreeding success of previously nutrient restricted dams, it negatively impacted calf pre-weaning growth, reducing calf weaning weight by 28 kg. Further investigation into physiological mechanisms involved in the decreased milk production due to late gestational nutrient restriction beyond mammary blood flow is warranted. How insults to milk production and mammary blood flow after gestational undernutrition during the first parity may affect lactational performance in later parities should also be explored.

## Abbreviations

ADF: acid detergent fiber
BCS: body condition score
BW: body weight
CP: crude protein
DDGS: dried distillers' grains with solubles
DM: dry matter
ME: metabolizable energy
MP: metabolizable protein
NDF: neutral detergent fiber
